# Athlete Identity, Resilience, Satisfaction with Life and Well-Being of Para Badminton Players: A Multinational Survey

**DOI:** 10.21315/mjms2024.31.3.13

**Published:** 2024-06-27

**Authors:** Siew Li Goh, Ngan Ling Wong, Poh Li Lau, Garry Kuan, Li Chongwei, Emily Kui Ling Lau

**Affiliations:** 1 Sports and Exercise Medicine Research and Education Centre, Centre for Epidemiology and Evidence-Based Practice, Faculty of Medicine, Universiti Malaya, Kuala Lumpur, Malaysia; 2 Department of Asian and European Languages, Faculty of Languages and Linguistics, Universiti Malaya, Kuala Lumpur, Malaysia; 3 Department of Educational Psychology and Counselling, Faculty of Education, Universiti Malaya, Kuala Lumpur, Malaysia; 4 Exercise and Sports Science Programme, School of Health Sciences, Universiti Sains Malaysia, Kelantan, Malaysia; 5 Department of English Language, Faculty of Languages and Linguistics, Universiti Malaya, Kuala Lumpur, Malaysia

**Keywords:** adaptive sports, para-athletes, quality of life

## Abstract

**Objective:**

To explore regional differences (i.e. Europe, Asia and others) in the well-being of para-athletes and its potential psychosocial determinants, including the Athletic Identity Measure Scale (AIMS), the Brief Resilience Scale (BRS) and the Satisfaction with Life Scale (SWLS).

**Methods:**

The study was a cross-sectional survey using data from multinational badminton federations. The study participants were athletes registered in the Para Badminton Classification Master List of the Badminton World Federation (BWF). The main study outcome is the WHO Quality of Life-Disability Questionnaire (WHOQOL-DIS).

**Results:**

There were 1,385 (aged 36 years old, IQR 18 years old) registrants on the master list. Respondents totaled 170. Only 137 (65% were males) were included in the analysis after excluding those with missing data (Europe 40%, Asia 30%, others 30%). Following the results of factor analysis, the original Athletic Identity Measure Scale (AIMS) was separated into self-identity (SI) and AIMS-modified. SI, AIMS-modified, the BRS and the Satisfaction with Life Scale (SWLS) were all scored above average. The AIMS-modified scores of Europeans were significantly lower than those of other non-Asians (U = 757.000, *P* < 0.05). BRS was statistically higher among those with acquired disabilities (median: 3.33) compared to those with congenital disabilities (median: 3.0) (U = 1,717.000, *Z* = *−*2.711, *P* < 0.05) and among Europeans (median: 3.3) compared to Asians (median: 3.0) (U = 704.500, *P* < 0.05). The regression model explained 32% of the variability in quality of life (QOL) with five significant predictors. The SWLS (*β* = 0.307, *P* = 0.01), BRS (*β* = 0.269, *P* = 0.01), full-time employment (*β* = 0.191, *P* = 0.05) and being female (*β* = 0.162, *P* = 0.05) all had a positive effect on QOL, but not the AIMS (*−*0.228, *P* = 0.05).

**Conclusion:**

The results show that the athletes’ resilience, satisfaction with life and identity vary across regions. Furthermore, satisfaction with life, employment and gender were found to be significant predictors of athletes’ QOL.

## Introduction

Since sports are a form of physical activity, they could offer the same physical and mental health benefits that are often related to exercise. Unlike exercise however, sports are primarily undertaken for competitive purpose, creating opportunity to gather people from different social and cultural background who wish to pit their physical prowess and skills. Recognising that sports offer a great platform to promote social inclusivity, Ludwig Guttman—a Jewish neurologist—introduced sports for the rehabilitation of casualties of World War II with spinal injuries. The initiative which started as a competition for paraplegic archer, went on to become the Paralympic Games as more sports were included (1).

People with disabilities can reach their full potential, learn new skills for life, gain confidence, and improve their quality of life (QOL) through sports (2). The environment of competitive sports nurtures qualities that drive performance and athleticism. Eventually, many who compete will identify themselves based on their athletic role and develop an athletic identity. Athletic identity is generally regarded to be positive because it portrays attributes of dedication, motivation and resilience (2). Resilience generally refers to the ability to overcome stress or adversity, giving rise to positive psychological outcome (3). It has been observed that disabled adults who participate in sports are as resilient as healthy adults, and more resilient than disabled adults who do not participate in sports (4).

On the other hand, it can also be argued that the well-being of athletes, especially elite athletes, may be adversely affected by the intense competitive environment of high-performance sports. They need to cope with high expectations of coaches and are at higher risk of training burnout and injuries (5, 6). Although these challenges are similar in disabled and abled-bodied athletes, athletes with disability face additional issues. Compared to able-bodied athletes, para-athletes have been reported to experience lower self-acceptance, peer discrimination, and restricted training and coaching resources (7). On the other hand, compared to the general population, para-athletes had more stress because they were worried about their sports performance, doping in sports and getting ready for retirement (8).

Considering the complex interaction of multiple factors both inside and outside of sports that would potentially affect the well-being of para-athletes, this study was undertaken to assess the general well-being of Para badminton players from different regions and to investigate the relationship between their well-being and psychosocial factors, including those related to the levels of athlete identity, life satisfaction and resilience. Apart from meeting the need for multinational data, this survey was also undertaken in response to the call by Badminton World Federation (BWF), the international governing body of badminton, to bridge the gap in research on badminton players with disabilities. Although competitive sports for disabled athletes had been in existence for more than 100 years (9), badminton was not internationally contested until 30 years ago. It was brought under the governance of BWF only in the last 10 years (10, 11).

## Methods

### Participants

Irrespective of their disability or level of participation, all Para players who have registered under the BWF’s Para Badminton Classification Master List are eligible for the study. The players were reached through their respective national badminton federations. Athletes who were unable to understand the questionnaires either due to intellectual impairment or poor command of the English language and who were without a translator were excluded from this study.

### Outcome Measures

Four questionnaires comprising of Athletic Identity Measure Scale (AIMS), Satisfaction with Life Scale (SWLS), Brief Resilience Scale (BRS) and WHOQOL Disability module (WHOQOL-DIS) were used as surrogate measures of level of athlete identity, life satisfaction, resilience and well-being, respectively. These questionnaires were distributed only in English because it was not possible to obtain validated set of questionnaires in different languages for each of the instruments.

#### Athletic Identity Measure Scale

This is the commonest and most widely validated tool for measuring athlete identity—i.e. measuring the level at which the athletic role contributed to the self-concept of the respondents (12). However, validity of AIMS appears to differ between different athlete populations with some researchers adopting the 7-item and others the 10-item version of the questionnaire (13, 14). In this study, we administered the 10-item model proposed by Martin et al. (15) with each item being scored on 1–7-point Likert scales with higher score representing stronger athlete identity (16). The questionnaire, as originally proposed, was shown to assessed four factors: i) social identity (the awareness of how society perceive the participant’s role as athlete), ii) self-identity (SI) (personal awareness of the participant’s role as athlete), iii) negative affectivity (negative emotional response to adversities) and iv) exclusivity (how exclusive is their athletic role in comparison to other roles) (15).

#### Satisfaction with Life Scale

To assess the respondents’ subjective sense of well-being, a simple 5-item SWLS was used, following the proposed 1–7-point Likert scale (17). Life satisfaction is a construct commonly linked to one’s evaluation of well-being as a whole. The higher the aggregate score, the greater is the life satisfaction. Although widely used, there is also evidence to suggest that validity of SWLS questionnaire is affected by group differences (18).

#### Brief Resilience Scale

We used the BRS that contained six questions scored on 5-point Likert scale to gauge the adaptability of the players to adverse environment and life challenges (19). The total score is positively correlated to resilience level.

#### WHOQOL-DIS

The disability module from the WHOQOL questionnaire specifically assesses the impact of disabilities on the well-being of the respondents. It is supplementary module to the existing WHOQOL tools (20). Examples of items found in WHOQOL-DIS are: “do you feel that some people treat you unfairly” and “do you need someone to stand up for you when you have problems”. There are 13 items in total and each is scored on a 5-point Likert scale. Scoring is obtained by averaging the scores from each item with higher value representing positive outcomes.

### Data Collection

The questionnaires were disseminated online to all national badminton federations affiliated to BWF over 6 months between mid-February 2020 to mid-August 2020. Coaches or personnel in charge of disabled badminton were requested to pass on the call for participation to their respective national players of all categories of disability. The coaches or personnel in charge were also requested to assist in translating the questions to those who do not understand English. Additional demographic and socioeconomic data (i.e. gender, country or origin, type of disability, education, employment status and competition experience) were also collected.

### Statistical Analysis

Considering that the performance of questionnaire validity is dependent upon population groups, a factor analysis was performed with Oblimin rotation prior to descriptive analysis. The 21 items from AIMS (10 items), SWLS (5 items) and BRS (6 items) were evaluated using the Kaiser-Meyer-Olkin value and Barlett’s test of sphericity (21). Internal consistency of all factors or constructs, including WHOQOL scale was examined using Cronbach’s alpha.

Unless the Kolmogorov-Smirnov test supported data normality, data from all four instruments were summarised or scored as continuous variable and presented as median and interquartile range (IQR). Differences between groups were evaluated using non-parametric tests: Kruskal-Wallis and Mann-Whitney U for categorical predictors (e.g. gender and education level) and Spearman’s rho for continuous predictor (e.g. years of competition).

Multiple regression was used to examine the ability of AIMS, SWLS, BRS and other demographic variables to estimate the QOL (WHOQOL-DIS) (22) of Para badminton athletes. The minimum sample size for power analysis is 84 to gain the power of 0.80 (based on R^2^ = 0.20) (23).

For variables where the data for one or more of the subgroups appear to be imbalanced, the subgroups would be simplified before analysis. The sociodemographic data was thus categorised as follows for analysis purpose: -

- Region : Asia, Europe, others
- Gender : Male, Female
- Sports classification : Wheelchair-bound (WH1—severe impairment, WH2—minor impairment), Standing (SL3—lower limb impairment, SL4—lower limb impairment minor, SL5—upper limb impairment), Short stature (SH6)
- Nature of disabilities : Congenital, Acquired
- Employment status : Full-time (Fixed paying job, self-employed), not full-time (part time job, others: e.g. social benefit, pension, unemployed)
- Education level : Secondary and lower (primary, secondary), tertiary and others (diploma, first degree, masters)

Correlation between outcomes were assessed using only completed data sets (i.e. responses had been recorded for all four questionnaires).

Statistical analyses were carried out using SPSS Statistics version 27.0 (IBM, New York). The significant level was set at *P*-value < 0.05 except in bivariate analysis where Bonferroni correction was applied. Questionnaires with missing response were excluded from the analysis.

## Results

There were 1,385 athletes (aged 36 years old, IQR 18 years old) registered under BWF’s Para Badminton Classification Master List. A total of 170 athlete responded to the survey of which 137 participants (65% of the participants were males) managed to complete all four questionnaires. Athletes from European countries constituted 37% of the respondents, Asia 32% and other continents (i.e. Africa, North America, South America, Oceana) 29%.

Description of the 137 responses used for analysis is shown in Table 1.

### Factor Analysis

The Kaiser-Meyer-Olkin value was 0.78, exceeding the recommended value of 0.6 (Kaiser 1974) and Bartlett’s test of sphericity (Bartlett 1954) reached statistical significance, supporting the factorability of the correlation matrix. Initial factor analysis revealed low factor loadings for two items of athletic identity measurement (Items 3 and 8) and three items of BRS (Items 1, 3 and 5). These items were hence removed. The analysis of retained items revealed the presence of four factors, each with Eigenvalues exceeding 1 (Table 2). Our findings support AIMS being a 2-factor model where the original SI construct is retained with Items 1 and 2 (Factor 4). Other constructs (i.e. social identity, negative affectivity, exclusivity and one unexplained domain) are now merged as Factor 1. Items from SWLS and BRS have retained their unidimensional construct as Factor 2 and 3, respectively. Together, these four factors explained a total of 65.5% of the variance (refer to Table 2) with Factor 1 being the largest explanatory variable (29.8%).

Internal consistency for all factors or constructs, including WHOQOL Scale, was examined using Cronbach’s alpha and all the constructs were above the recommended value of 0.70 (Table 3).

The result of this factor analysis is used to guide the subsequent inferential analyses involving AIMS and BRS. To differentiate the newly identified construct, Factor 1 would be referred to as AIMS-modified, Factor 2 would be referred to as SWLS, Factor 3 as BRS-modified and Factor 4 as SI.

As summarised in Table 3, the score for each item across all self-reported outcomes were above average: SI 6.1 (standard deviation [SD] 0.9); AIMS-modified 5.3 (SD 1.2); SWLS 4.9 (SD 1.1); BRS-modified 3.2 (SD 0.8) and WHOQOL-DIS 3.9 (SD 0.7)

### Bivariate Analysis

#### Regional Difference

SI, AIMS-modified and BRS-modified were significantly affected by region (Table 4). Mann-Whitney U tests were used to follow up these findings. A Bonferroni correction was applied to adjust for multiple testing. Therefore, all effects are reported at a 0.0167 level of significance.

There was no statistical difference in regional score for WHOQOL-DIS measure. Athletes reported an average score of 4 on the 5-point Likert scale. European players were no different than Asian players for SI measure but scored significantly lower ratings in SI compared to players from other regions (U = 687.000, *P* < 0.05) and in AIMS-modified compared to players from other parts of the world (Asia: U = 631.500, *P* < 0.01; other: U = 757.000, *P* < 0.05). Also, while others had the highest mean resilience score (3.667), it was only significantly different between Europeans and Asians (U = 704.500, *P* = 0.05).

#### Gender

The median scores for SI, modified identity and satisfaction with life were highest among females, but the median score for resilience was the same for both males and females (Table 5).

#### Nature of Disability

For all outcomes, no difference was found between wheelchair, standing or short stature players. However, there is significantly higher resilience among those with acquired disabilities (median = 3.333) compared to those with congenital disabilities (median = 3.000) (U = 1,717.000, *Z* = −2.711, *P* < 0.05).

#### Social Factors

Employment, education status and number of competitive years did not contribute to any difference in all the self-reported outcomes.

### Regression Analysis

Eleven independent variables (SI, AIM2, SWLS, BRS2, region, gender, classification, nature of the disability, source of income, higher education level and the number of years of competition at national or international level) were included in the regression model. The sample size is deemed adequate to test the coefficients of 11 variables which according to Tabachnick and Fidell (24), the minimum required would be 115 (104 + *m*, where *m* = the number of independent variables). Preliminary analyses were conducted to ensure no serious violation of the assumptions of normality, linearity, multicollinearity and homoscedasticity.

This 11-predictor model significantly estimated 32.5% of the variance in the well-being of Para badminton players. Five factors (SWLS, BRS-modified, AIMS-modified, gender and source of income) were found to be independent predictor of WHOQOL-DIS with SWLS recording the highest beta value (*β* = 0.307, *P* < 0.05), and then followed by BRS-modified (*β* = 0.269, *P* < 0.05), AIMS-modified (*β* = −0.228, *P* < 0.05), source of income (*β* = 0.191, *P* < 0.05) and gender (*β* = 0.162, *P* < 0.05) (Table 6). In brief, greater satisfaction with life, greater resilience, full-time employment and female predicts better well-being. Whereas higher level of athlete identity appeared to have negative impact on well-being.

## Discussion

This is one of the few large-scale studies that looks at how many athletes from different regions of the world take part in Para badminton sports. We aimed to evaluate the well-being of Para badminton players and explore its association with sports-related psychosocial factors (i.e. athlete identity and resilience). Other than regional difference in athletic identity and resilience measures, we had identified potential predictors of better well-being (i.e. high SWLS, high BRS, lower AIMS-modified, full-time employment and female).

The key strength of this study lies in the scale of the survey and the comparatively large number of responses received in a single Para sport (7, 25). An advantage of single sport study is minimised heterogeneity caused by the diversity in governance and practices of different sports. Additionally, the active involvement of BWF in informing the study design and in facilitating data collection helped to optimise the yield of the survey. With the questionnaire instituted out-of-competition, this ensured the athletes were not under duress or pressure to conform to stereotype responses. Lastly, considering that there is relative lack of specific psychosocial tools for Para athletes, we examined the internal consistency of all factors or constructs of the questionnaires used prior to the analysis. Apart from AIMS, the construct measured by SWLS and BRS have been confirmed to follow the unidimensional model as were originally intended.

Unlike Martin et al. (15), our study suggested that the original AIMS questionnaire was measuring only two factors instead of four in this population of Para badminton players. One of the factors corresponded to the original construct of SI (i.e. AIM01 and AIM02). The second construct was formed by the remainder items (i.e. AIM04–AIM07, AIM09–AIM10), which had originally been shown to measure three separate constructs. While the first construct of SI did not impart significant effect on athlete well-being, the second construct (i.e. AIMS-modified) was found to correlate negatively with WHOQOL-DIS. Although strong athletic identity may confer favourable impact on athlete achievements, it does not necessarily produce better health outcomes. It has been reported that athletes with strong athlete identity were at higher risk of developing depression after injury and after retirement (26). The negative correlation observed in this study suggests that the athletes are likely to face difficulties in adapting to live outside of competitive sports and also possibly feeling overwhelmed by the expectations imposed on them.

In contrast to AIMS, correlation between SWLS, BRS and WHOQOL-DIS were positive. These observations agree with many other earlier studies, supporting the benefits of higher SWLS, BRS and overall well-being of athletes (27). Although a causal relationship between resilience or life satisfaction and QOL could not be established from our study, an association provides sufficient justification to promote positive emotions, especially resilience among Asian athletes. Although SWLS was comparable across regions, there is significant regional discrepancy in BRS with Asians scoring the lowest. The reason for the lower resilience measures among Asians players is unclear. Resilience level is related interaction between cultural identity, social support, personal experience and life adversities (28). Further studies are needed to elucidate the factors responsible for the lower resilience among Asian players.

Another crucial aspect for ensuring higher QOL among these athletes is financial stability. Athletes with full-time employment reported better WHOQOL-DIS measures compared to those without. This finding supports the need for governing agencies and relevant stakeholders to formulate strategies related to employment for the athletes, preparation for retirement and instituting better support for dual career athletes. The importance of athletes to have balanced success in education/vocation and sports is likely to contribute significantly to their QOL. Considering that seeking employment is challenging for people with disability, effort to address discrepancies in renumerations and support system between disabled and able-bodied athletes is a much welcome move towards improving the QOL for disabled athletes (29).

Other than the inherent limitations associated with surveys (e.g. missing data and recall biases), this study had additional challenge in ensuring that the questionnaires are cross-culturally valid. Questionnaires in English was used and much reliance was placed on the translators and non-native English speakers to provide appropriate response. Another limitation is the seemingly low response rate of 10% of target population. However, this may be attributed by uncensored records, as athletes who are no longer competing may still be retained in the registry. As indicated by the low model fit of 32% in the regression model, predictors of well-being among Para badminton players would warrant further studies as it is a complex construct underpinned by multiple intrinsic and extrinsic factors.

## Conclusion

In the present study, the SI, AIMS-modified, BRS and SWLS were shown to have adequate psychometric properties among para-badminton athletes using exploratory factor analysis (EFA). The findings demonstrate that regional differences exist in the athletes’ resiliency, life satisfaction and sense of identity. Additionally, it was discovered that the QOL of athletes was significantly predicted by life satisfaction, occupation and gender. Further studies are warranted to better elucidate these relationships and identify ways to better support Para athletes in the spirit of an inclusive society. Rigorous evaluation and identification of appropriate questionnaires and measurement tools for this population should also be equally emphasised.

## Figures and Tables

**Table 1:** Demographic and description of complete responses analysed (*N* = 137)

Demographic	
**Continent** # **:**	
Europe	55 (40.1)
Asia	41 (29.9)
Others	41 (29.9)
**Gender (Male/Female)**	89/48
**Classification**	
Wheelchair	57 (41.6)
WH1	37 (27.0)
WH2	20 (14.6)
Standing	80 (58.4)
SL3	21 (15.3)
SL4	26 (19)
SL5	19 (13.9)
SH6	14 (10.2)
**Disability**	
Acquired	72 (52.6)
Congenital	65 (47.5)
**Employment**	
Full-time	61 (44.5)
Fixed pay	43 (31.4)
Self-employed	18 (13.1)
Not full-time	76 (55.5)
Part time	40 (29.2)
Others	36 (26.3)
**Education**	
Secondary and below	56 (40.9)
Primary	4 (2.9)
Secondary	52 (38)
Tertiary	81 (59.1)
Diploma, University, College	78 (56.9)
Others (unclear)	3 (2.2)
**Competing experience, years (median, IQR**)	4 (6)

Notes:

# Numbers are count; percentages are in parenthesis (unless indicated otherwise)

**Table 2:** Pattern matrix for factor analysis with Oblimin rotation of four factors solution

Items	Factor 1	Factor 2	Factor 3	Factor 4	Communalities
AIMS-merged					
AIM05	**0.882**	−0.043	0.116	−0.033	0.746
AIM04	**0.861**	−0.080	0.054	0.086	0.769
AIM06	**0.729**	0.015	0.087	−0.034	0.530
AIM09	**0.618**	0.110	−0.137	0.065	0.444
AIM10	**0.587**	−0.077	−0.112	−0.009	0.393
AIM07	**0.506**	0.202	−0.148	−0.136	0.494
SWLS					
SWLS02	−0.140	**0.778**	0.083	−0.052	0.636
SWLS04	−0.036	**0.736**	−0.029	0.064	0.507
SWLS03	−0.075	**0.736**	0.075	−0.065	0.578
SWLS05	0.172	**0.577**	0.015	0.060	0.367
SWLS01	0.083	**0.528**	−0.084	−0.182	0.415
BRS					
BRS04	−0.013	0.126	**0.736**	0.016	0.579
BRS06	0.050	−0.007	**0.691**	0.001	0.459
BRS02	−0.030	−0.043	**0.684**	−0.010	0.477
AIMS-SI					
AIM01	−0.062	0.007	0.013	**−0.953**	0.861
AIM02	0.230	0.042	−0.030	**−0.616**	0.590
Eigenvalues	**4.772**	**2.952**	**1.696**	**1.061**	
% of variance explained	29.827	18.451	10.598	6.632	

Note: Extraction method: principal axis factoring; Rotation method: Oblimin with Kaiser normalisation; Rotation converged in 5 iterations; Major loadings for each item are bolded; AIMS = Athletic Identity Measure Scale (10 items, 1–7 Likert); SWLS = Satisfaction with Life Scale (5 items, 1–7 Likert); BRS = Brief Resilience Scale (6 items, 1–5 Likert)

**Table 3:** Results of measurement assessment

Constructs/Factors	Items	Mean	SD	IQR	Cronbach’s alpha
SI	2	6.120	0.919	1.000	0.798
AIMS-modified	6	5.251	1.201	1.500	0.861
SWLS	5	4.909	1.136	1.500	0.807
BRS-modified	3	3.173	0.815	1.000	0.746
BWF Para badminton athlete	13	3.906	0.658	0.923	0.856
Well-being (QoL)					

**Table 4:** Pattern matrix for factor analysis with Oblimin rotation of four factors solution

Region	Region	Median	*n*	Kruskal-Wallis H	df	*P*-value
SI	Asia	6.000	41	11.941	2	0.003
	Europe	6.000	55			
	Others	6.500	41			
AIMS-modified	Asia	5.833	41	14.688	2	0.001
	Europe	5.167	55			
	Others	5.833	41			
SWLS	Asia	5.000	41	0.125	2	0.939
	Europe	5.000	55			
	Others	5.200	41			
BRS-modified	Asia	3.000	41	11.720	2	0.003
	Europe	3.333	55			
	Others	3.667	41			
WHOQOL-DIS	Asia	3.846	41	2.500	2	0.287
	Europe	4.077	55			
	Others	4.154	41			

**Table 5:** Mann-Whitney U test for SI, AIMS-modified, SWLS, BRS-modified and WHOQOL-DIS by gender

Constructs	Gender	Median	*n*	Mann-Whitney U	*Z*	*P*-value
SI	Male	6.000	89	1,738.500	−1.849	0.064
	Female	6.500	48			
AIMS-modified	Male	5.333	89	1,704.500	−1.950	0.051
	Female	5.750	48			
SWLS	Male	5.000	89	2,100.000	−0.163	0.871
	Female	5.200	48			
BRS-modified	Male	3.000	89	2,061.000	−0.342	0.733
	Female	3.000	48			
WHOQOL-DIS	Male	4.000	89	1,836.500	−1.353	0.176
	Female	4.077	48			

**Table 6:** The regression model for WHOQOL-DIS

Constructs/Variables	Unstandardised		Standardised coefficients	*P*-value	95% confidence interval for B	
	
	B	Std. Error	*β*		Lower bound	Upper bound
(Constant)	2.066	0.481		0.000	1.115	3.018
SI	0.110	0.067	0.154	0.101	−0.022	0.242
Athletic Identity	−0.125	0.051	−0.228	0.017*	−0.227	−0.023
Life satisfaction	0.178	0.047	0.307	0.000*	0.086	0.270
Resilience	0.217	0.067	0.269	0.002*	0.084	0.350
Asia (ref. others)	−0.060	0.147	−0.042	0.685	−0.350	0.231
Europe (ref. others)	−0.184	0.137	−0.138	0.181	−0.455	0.087
Female	0.222	0.112	0.162	0.049*	0.001	0.443
SL (ref. WH)	0.017	0.123	0.012	0.891	−0.227	0.261
SH (ref. WH)	0.238	0.145	0.155	0.103	−0.049	0.525
Nature of disability (ref. Congenital)	0.136	0.116	0.104	0.244	−0.094	0.367
Fixed income and self-employed (ref. Part-time and others)	0.252	0.106	0.191	0.019*	0.041	0.462
Tertiary and above (ref. Secondary and below)	−0.005	0.100	−0.004	0.959	−0.203	0.193
Number of years of competition at national/international level	0.005	0.008	0.048	0.555	−0.011	0.021
